# Ultraviolet Upconversion Emission of CaAl_2_SiO_6_ Polycrystals Doped with Pr^3+^ Ions

**DOI:** 10.3390/molecules30142944

**Published:** 2025-07-11

**Authors:** Karol Lemański, Nadiia Rebrova, Patrycja Zdeb-Stańczykowska, Przemysław Jacek Dereń

**Affiliations:** Institute of Low Temperature and Structure Research, Polish Academy of Sciences, ul. Okólna 2, 50-422 Wrocław, Poland; n.rebrova@intibs.pl (N.R.); p.zdeb@intibs.pl (P.Z.-S.); p.deren@intibs.pl (P.J.D.)

**Keywords:** luminescence, UVC, praseodymium, upconversion, polycrystals, aluminosilicates, spectroscopy

## Abstract

The spectroscopic properties of Pr^3+^ ions in the aluminosilicate matrix were investigated for the first time. Synthesis of CaAl_2_SiO_6_ (CASO) polycrystals doped with Pr^3+^ ions was carried out using the sol–gel method. The crystalline structures have been confirmed with XRD measurement. The absorption, excitation, emission spectra, and time decay profiles of the praseodymium (III) ions were measured and analyzed. It was found that upon excitation with visible light, this material exhibits emission mainly in the UVC region, via an upconversion emission process. The Stokes emission in the visible range is observed mainly from the ^3^P_0_ and ^1^D_2_ energy levels. The ^1^D_2_→^3^H_4_ emission is very stable even at very high temperatures. The studied aluminosilicate phosphors possess characteristics that confirm their potential in upconversion emission applications.

## 1. Introduction

The phenomenon of upconversion emission was first described by N. Bloembergen [1] and soon after by F. Auzel [2]. There are various upconversion mechanisms [3,4]. The probability of their occurrence is different, which is why their efficiency and frequency of occurrence are different. Most publications related to this phenomenon concern the conversion of infrared radiation into visible light, where Er^3+^-Yb^3+^ [5,6] or possibly Ho^3+^-Yb^3+^ [5,7], Tm^3+^-Yb^3+^ [8], Tb^3+^-Yb^3+^ [9], and also Pr^3+^-Yb^3+^ [10,11] ions are usually used, due to the appropriate matching of the energy levels of electrons on the 4f subshell and the effective energy transfer from Yb^3+^ ions to other lanthanide ions. This process can also concern the conversion of visible light, e.g., blue, into high-energy radiation in the ultraviolet range. This creates additional application possibilities due to the disinfecting properties of UV radiation, which destroys viruses and bacteria; mainly UVC [12] but also UVB [13] and even UVA [14,15] could be used for disinfection purposes.

Praseodymium ions are good candidates for various applications in the luminescence field, to generate visible light [16,17,18], and also for emitting UV radiation from the 5d level in suitable matrices [19,20], because this level is at a large distance from the lower levels, such as ^3^P_J_, so luminescence quenching via multiphonon relaxation processes [21,22] will not occur, even for matrices with high phonon energy. Not all luminescent materials are good candidates for generating UV radiation through the upconversion processes, especially in the high-energy UVC region (100–280 nm), which destroys germs. Effective UVC radiation for the Pr^3+^-doped samples, induced by the upconversion process, should occur from the 5d level of Pr^3+^ ions; therefore, the energy bandgap of these materials should be large enough for the 5d level to be situated inside this energy bandgap, not in the conduction band. The 5d-4f emission is allowed by the selection rule transition, so it will be characterized by good intensity and a short emission lifetime. The threat for efficient emission from the 5d level is the high 4f energy level ^1^S_0_, which, depending on the crystal matrix, is located inside the 5d band or slightly below this band, because the location of the 5d band can be different for the different hosts. Aluminosilicate CaAl_2_SiO_6_ is a good matrix for potentially efficient upconversion emission in the UVC region, because it has a high value of the energy bandgap, and also because the ^1^S_0_ multiplet is located above the minimum of the 5d band, so there will be no parasitic quenching processes of this emission in this case.

The investigated aluminosilicate CaAl_2_SiO_6_ has a sufficiently large energy bandgap, which will be described in detail later in the text. Moreover, it also has a ^1^S_0_ level located inside the 5d band, so it is a suitable candidate for obtaining efficient upconversion of emission in the UVC region. CaAl_2_SiO_6_ Yoshiokaite mineral structure was first collected from the Moon by the Apollo 14 crew in 1971 [23]. In the scientific literature, there are some papers describing the luminescent properties of CaAl_2_SiO_6_ [24,25,26,27,28], but still not all lanthanides have been doped into this host to check its spectroscopic characteristics.

In this work, we present novel results on the luminescent properties of Pr^3+^ ions in the aluminosilicate matrix CaAl_2_SiO_6_. Particular attention was given to the results concerning the rarely recorded conversion of visible light to emission in the UVC region.

## 2. Results and Discussion

**Figure 1:**
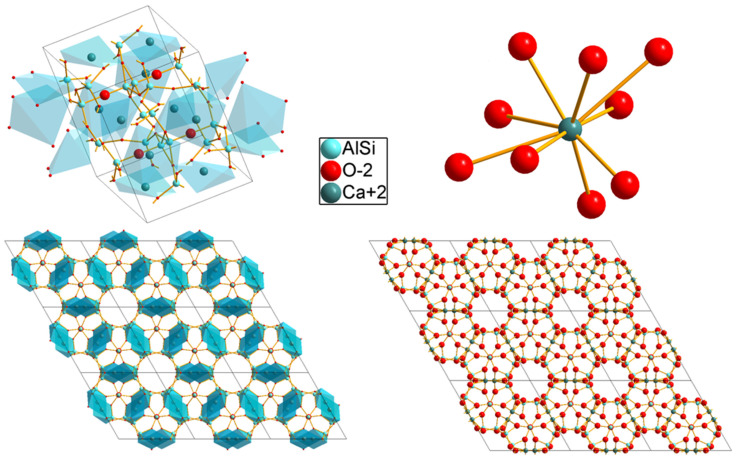
Visualization of the investigated aluminosilicate crystal structure (ICDD 01-080-1547).

The exact formula of the Yoshiokaite structure of the studied aluminosilicate is Ca_5.35_Al_10.7_Si_5.3_O_32_ [29]. Crystals possess a P-3 (147) trigonal space group, where the ICDD (International Centre for Diffraction Data) code is 01-080-1547 (Figure 1), and the corresponding number of the ICSD (Inorganic Crystal Structure Database) is 69380. Details regarding the structural properties of CASO aluminosilicate were given in a previously published work [25]; however, after a more detailed structural analysis, it was found that Ca^2+^ ions are coordinated by nine oxygen ions, where the bond length of each Ca^2+^-O^2-^ pair is equal to 2.375, 2.413, 2.417, 2.482, 2.492, 2.718, 2.726, 2.920, 2.921 [Å]. The ionic radius of Ca^2+^ ions in the coordination of IX ligands is equal to 1.32 Å [30]. The radius of Pr^3+^ ions (1.319 Å) perfectly matches the ionic size of Ca^2+^ in this coordination.

**Figure 2:**
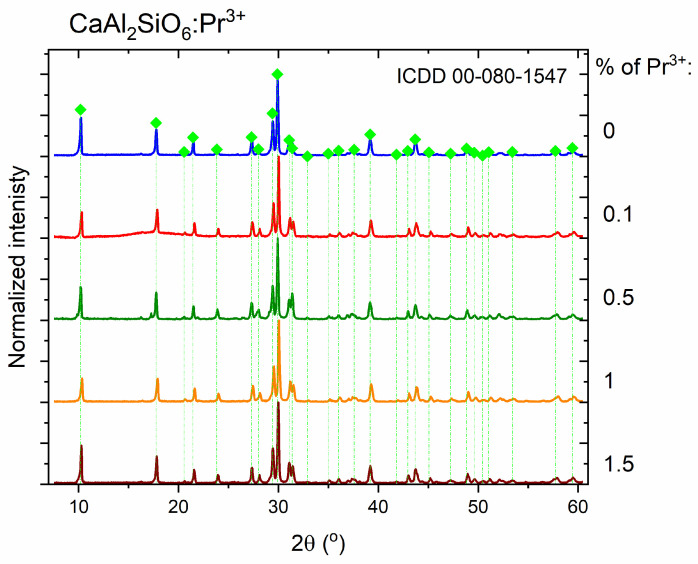
XRD patterns of the measured polycrystals of CaAl_2_SiO_6_.

The obtained diffractograms confirm the structure of the tested aluminosilicate for all samples (Figure 2). A slight, broad band with a center at about 15 degrees for the sample with the smallest amount of impurity, 0.1% Pr^3+^, indicates a slight share of the amorphous phase in the composition of this sample. The diffraction patterns of the investigated crystalline powders correspond to the standard from the crystallographic database with the number ICDD 00-080-1547.

**Figure 3:**
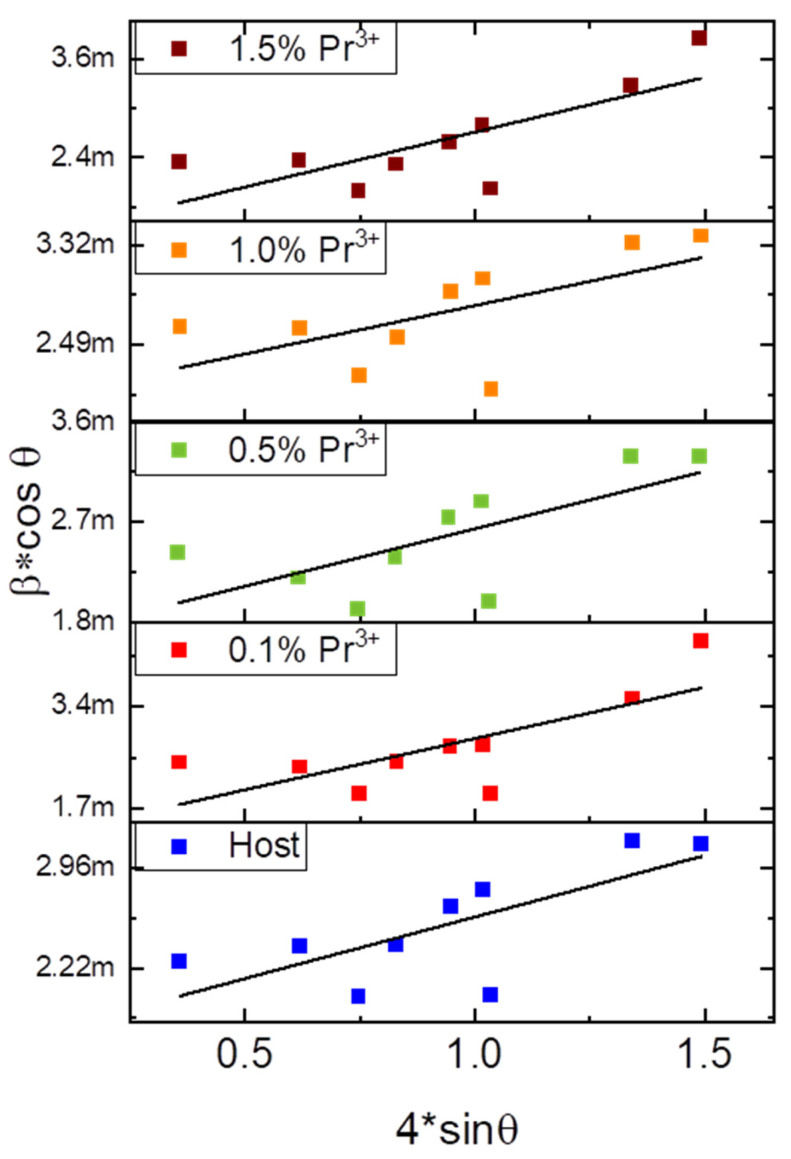
Plots of b*cosθ versus 4*sinθ for CaAl_2_SiO_6_ and CaAl_2_SiO_6_:Pr^3+^ particles.

The W-H analysis was performed by plotting 4sinθ on the *x*-axis against βcosθ on the *y*-axis for the six most intense diffraction peaks, as shown in Figure 3. The crystallite size and lattice strain were determined from the y-intercept and slope of the linear fit, respectively (Table 1). It should be noted that there is no observable dependence of crystallite size and lattice strain on the activator concentration.

With an increase in the concentration of doped Pr^3+^ ions and charge-compensating Na^+^ ions, there is a slight increase in the crystal lattice parameters (Table 1), which may be caused by the expansion of the volume of unit cells by the influence of larger sodium ions (Na^+^), whose ionic radius is 1.38 Å, which is larger than the radius of Ca^2+^ ions (1.32 Å). Based on the X-ray diffraction data, the crystallite size of particles was estimated using the Williamson–Hall (W-H) [31] plot and Scherrer method (SM) [32]. Since the SM does not account for deformations and lattice defects, the average particle size calculated by this method is smaller (~70 nm) compared to the particle size estimated by the W-H method (~90 nm).

For the chemical compound CaAl_2_SiO_6_, the energy gap size was previously estimated using the method based on the chemical formula of an inorganic compound [26,33,34]. According to the calculation presented in the recently published paper, the energy bandgap E_g_ = 7.68 ± 0.20 eV [26]. The size of the bandgap is large enough to accommodate the 5d level of Pr^3+^ ions. The ^1^S_0_ level is usually in the range of 46,000–47,000 cm^−1^ (217–213 nm), which in electron volts corresponds to a size of 5.70–5.83 eV, so this level also falls within the energy bandgap region.

**Figure 4:**
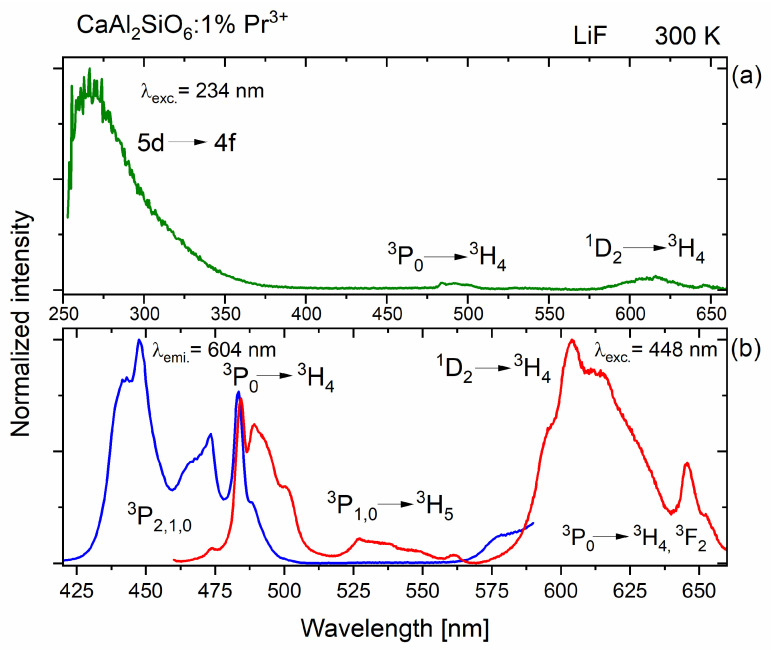
(**a**) 5d→4f emission spectrum of CaAl_2_SiO_6_:Pr^3+^ for λ_exc._ = 234 nm; (**b**) 4f→4f excitation and emission spectra of CaAl_2_SiO_6_:Pr^3+^ for λ_exc._ = 448 nm and λ_emi._ = 604 nm.

Figure 4a shows the excitation and Stokes emission spectra for a selected CaAl_2_SiO_6_ sample doped with Pr^3+^ ions. In order to precisely conduct studies in the ultraviolet range, measurements of the polycrystalline powder were performed using LiF glass, which is also transparent in the UV range. Measurements using non-synthetic quartz could be slightly disturbed by its absorption band in this range. The figure above shows the luminescence spectrum at excitation in the 4f-5d band at 234 nm. The emission spectrum of the 5d→4f band, centered at about 268 nm, is already very close to the excitation line, because it starts at about 250 nm. This band disappears in the visible region, at about 380 nm. The emission peaks from the ^3^P_0_ and ^1^D_2_ levels are much less intense. This indicates that the vast majority of the excitation energy is transferred directly to the 5d band, which does not lose much of this energy for transfer to the 4f praseodymium levels, because the ^1^S_0_ f-electron state is located above the lowest 5d state of the Pr^3+^ ions in this host. The wide energy range of the 5d level covers the ^1^S_0_ energy level. Thanks to this, the majority of the energy transferred to the 5d level is converted to the luminescence coming from this level, not non-radiatively to the ^1^S_0_ level. Therefore, the UVC emission from the 5d level of Pr^3+^ ions in the CaAl_2_SiO_6_ matrix is much more efficient.

In Figure 4b, the emission spectrum is presented, excited by a xenon lamp operating at a wavelength of 448 nm, matched to the Pr^3+^ level in the studied CASO matrix. This spectrum (red) is distributed over the excitation spectrum (blue), which is emitted when monitoring the emission at a wavelength of 604 nm, which defines the emission limits for ^1^D_2_→^3^H_4_ transitions. The visible ^3^P_0_ markings in these spectra are coincident with each other (Figure 4).

The emission bandwidth in the range of about 580–680 nm is quite significant. The most significant emission comes from the ^1^D_2_ level, but in a similar range, there may also be a ^3^P_0_→^3^H_6_ transition, which partially overlaps with the ^1^D_2_→^3^H_4_ emission. By using a femtosecond laser and a streak camera, it was possible to perform time-resolved measurements and separate these spectra.

**Figure 5:**
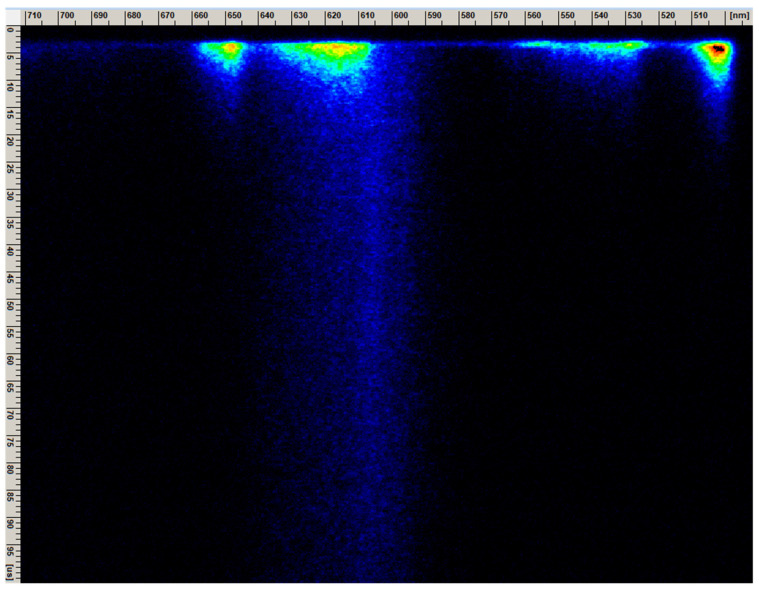
The image from the streak camera of CaAl_2_SiO_6_:Pr^3+^, visualizing the short- and longer-living emission from ^3^P_J_ and ^1^D_2_ energy levels, respectively (*Y*-axis represents time in µs and *X*-axis represents wavelength in nm).

Figure 5 shows an image obtained directly from the streak camera, with visible areas reflecting the luminescence intensity. The *X*-axis shows the wavelength region on the nanometer scale. The *Y*-axis shows time in microseconds. In addition to the short-lived luminescence from the ^3^P_0_ level, there is also visible longer-lived emission from the ^1^D_2_ energy level.

**Figure 6:**
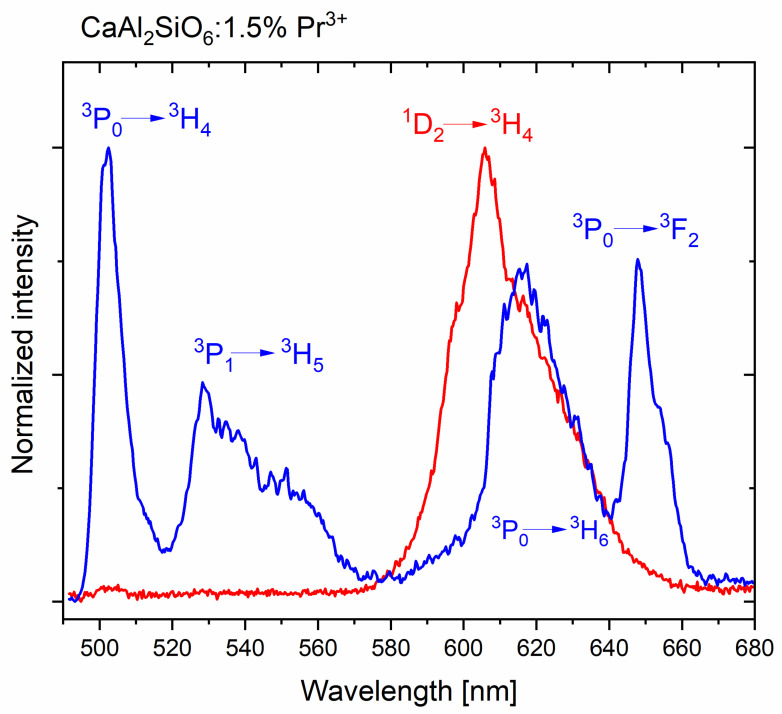
Emission spectra obtained from the streak camera of a femtosecond laser, with the separated emission from ^3^P_J_ and ^1^D_2_ levels of CaAl_2_SiO_6_:Pr^3+^.

The emission spectrum of CASO:Pr^3+^ (see Figure 6) was obtained from the measurements using a femtosecond laser streak camera, based on Figure 5, by using the program for processing spectra. In Figure 6, the blue color indicates the emission from the short-lived ^3^P_0_ (part) and ^3^P_1_ levels, while the red color indicates the longer-lived, spin-forbidden emission from the ^1^D_2_ level. (The streak camera covered an incomplete portion of the visible spectrum, so the ^3^P_0_→^3^H_4_ transition was only partially shown.) It is clearly visible that the ^3^P_0_→^3^H_6_ and ^1^D_2_→^3^H_4_ emissions overlap, which is commonly found in the luminescence of Pr^3+^ ions. By measuring the luminescence decay times or time-resolved spectra (Figure 6), it is possible to separate these emissions.

**Figure 7:**
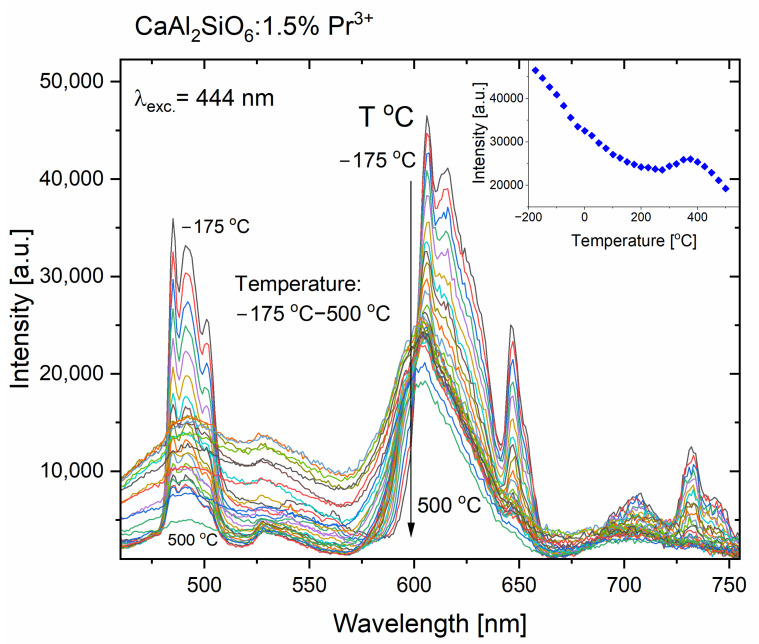
Temperature-dependent emission spectra of the CaAl_2_SiO_6_:Pr^3+^ polycrystals. Inset presents the intensity values of the ^1^D_2_→^3^H_4_ emission in CaAl_2_SiO_6_:Pr^3+^, changing with the increasing temperature.

Figure 7 shows the dependence of praseodymium (III) emission in CASO polycrystals for the visible range, depending on the temperature. The main luminescence from the ^1^D_2_ level to the ground state ^3^H_4_ does not completely quench, even at a very high temperature of 500 °C, and retains over 40% of its highest intensity recorded for −175 °C, while the emission from the ^3^P_0_ level is almost completely extinguished (see Figure 7). Moreover, it is interesting that the decrease in emission intensity possesses abnormal characteristics, due to the certain increase in the temperature range of 275–450 °C, where the maximum of this increase is for the temperature of 375 °C, which on the energy scale corresponds to the value of 450 cm^−1^. A probable explanation for this phenomenon is the intensification of cross-relaxation processes according to the scheme (^3^P_1_, ^3^H_4_)→(^1^D_2_, ^3^H_6_), due to the appropriate energetic matching of these levels. This is because the increased temperature allows for a larger population of electrons in the ^3^P_0_ excited state to reach the slightly higher energy of the ^3^P_1_ level. Looking back at the excitation spectrum for CaAl_2_SiO_6_:Pr^3+^ (see Figure 4), the ^3^H_4_→^3^P_0_ excitation peak has a maximum at 483 nm, which corresponds to an energy value of 20,704 cm^−1^.

On the other hand, the ^3^H_4_→^3^P_1_ transition was recorded at a wavelength of 473 nm (i.e., 21,142 cm^−1^). Therefore, it can be assumed that the energy difference between these levels is 438 cm^−1^; thus, it is almost resonantly matched to the temperature of 375 °C (450 cm^−1^), for which there is a significant increase in the occupancy of the ^3^P_1_ level from the ^3^P_0_ level. Therefore, the above-mentioned cross-relaxations occur, which additionally occupy the ^1^D_2_ energy level; thus, the emission from this level increases. Moreover, for this higher temperature, the ^1^D_2_ level could be additionally occupied from the ^3^P_0_ level by the multiphonon relaxation process. For higher temperatures, the cross-relaxation process (^1^D_2_, ^3^H_4_)→(^1^G_4_, ^3^F_4_) which quenches the emission from the ^1^D_2_ level [35] is more probable, due to the better energy matching for the phonon-assisted cross-relaxation process.

**Figure 8:**
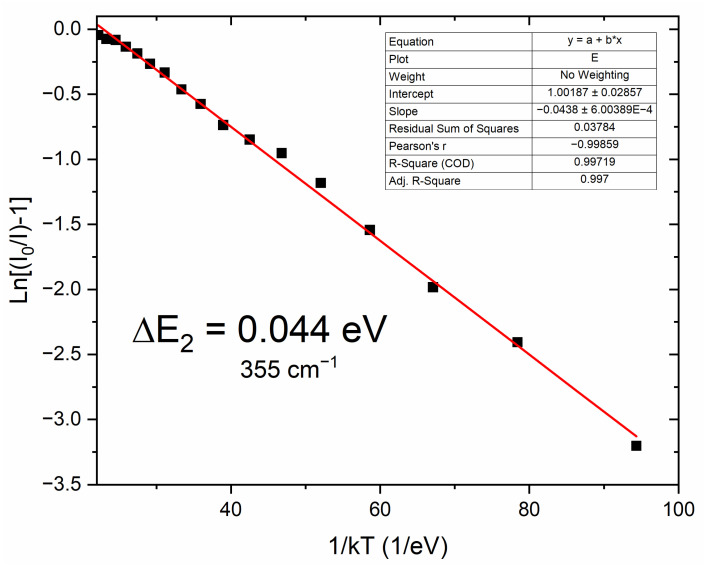
The activation energy of the thermal quenching, fitted using the Arrhenius equation for CaAl_2_SiO_6_:1.5% Pr^3+^ polycrystals.

Thermal quenching was described as a thermally activated process with an activation energy Δ*E*, which was performed by fitting the Arrhenius equation [36,37]:

(1) $$I\left(T\right)=\frac{I_{0}}{1+Ae^{-∆E/kT}},$$

where *I*_0_ is the initial emission intensity of the CaAl_2_SiO_6_:Pr^3+^ at the lowest temperature of −175 °C, *I*(*T*) is the intensity at different temperatures, *A* is a constant, *k* is the Boltzmann constant, and *T* is the temperature of the sample. Figure 8 presents the fit line of the thermal quenching emission model plotted as *Ln*((*I*_0_/*I*) − 1) against 1/*k*. Based on the Arrhenius equation (Equation (1)), the value of the activation energy for thermal quenching Δ*E* of this compound was calculated to be 0.044 eV, which gives the energy value of 355 cm^−1^. This is sufficient for electrons to move from the ^3^P_0_ to the ^3^P_1_ energy level, which quenches the emission coming from the ^3^P_0_ level.

**Figure 9:**
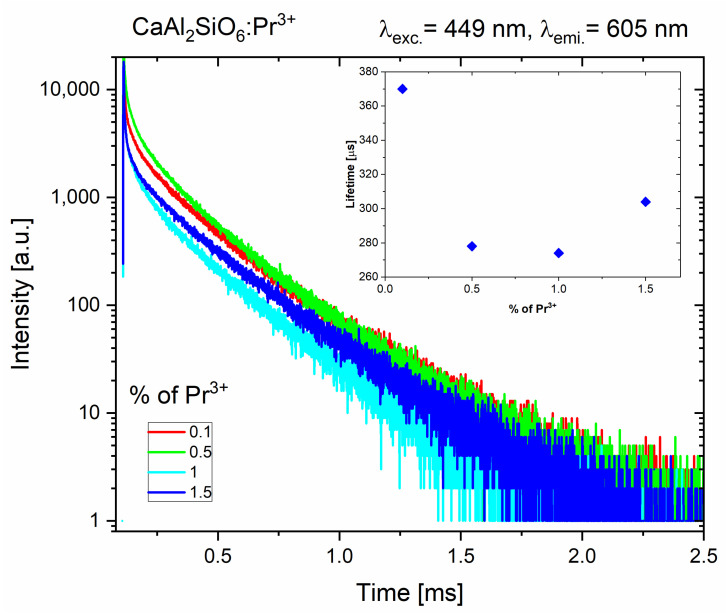
Luminescence decay curves and the obtained emission lifetimes values (inset) of the ^1^D_2_→^3^H_4_ emission in CaAl_2_SiO_6_:Pr^3+^.

The results of the luminescence lifetime kinetics measurements are presented in Figure 9 for the ^1^D_2_ emitting level of Pr^3+^. The luminescence decay curves have a non-exponential character. The average emission lifetime was determined using the integral formula [38]. The obtained results of ^3^P_0_→^3^H_4_ emission in CaAl_2_SiO_6_:Pr^3+^ are about 5–6 microseconds for all samples. The longest ^1^D_2_→^3^H_4_ emission lifetime of 370 milliseconds is possessed by the sample with the lowest concentration of Pr^3+^ ions, 0.1%. Samples containing higher Pr^3+^ concentrations have smaller, similar luminescence lifetime values. This may be due to the valence mismatch between Pr^3+^ and Ca^2+^ ions, and non-radiative processes between Pr^3+^ ions, such as energy migration and cross-relaxation processes, leading to the concentration quenching, even for relatively low amounts of Pr^3+^ dopant.

**Figure 10:**
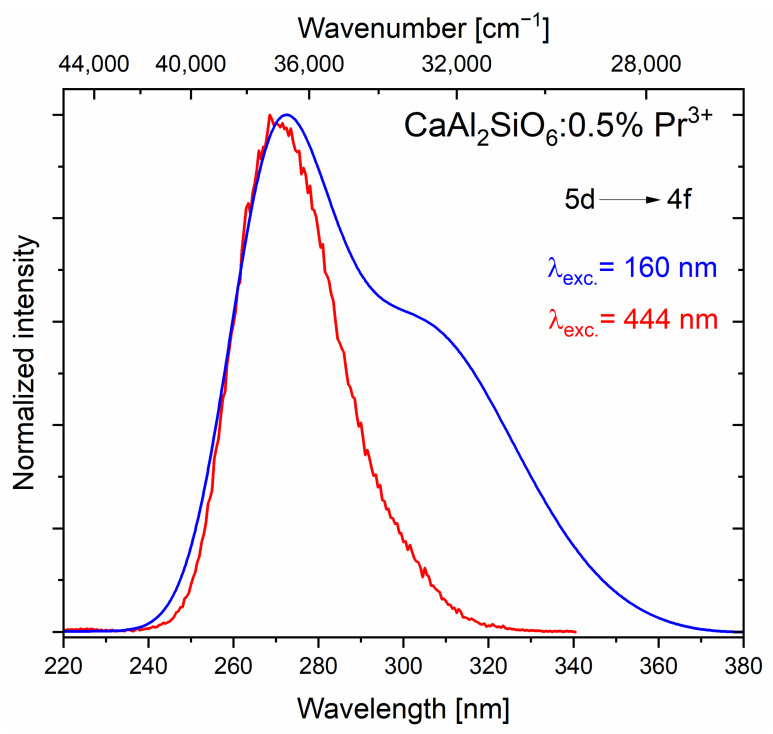
The 5d→4f emission in CaAl_2_SiO_6_:Pr^3+^, obtained through the Stokes (blue spectrum) and anti-Stokes (red spectrum) processes.

The two emission bands visible in Figure 10 represent emission in the UVC region, up to 280 nm, partly also in the UVB range, between 280 and 315 nm, and also a small part in the UVA range, which is from 315 to 400 nm. The broader spectrum, in the range of about 240–370 nm, consisting of two broad bands, was excited by radiation of a deuterium lamp with a wavelength of 160 nm.

The second, narrower emission band in the range of 240–330 nm visible in the figure was recorded using a blue laser operating at 444 nm. These spectra are identical, and the visible differences result from the use of different detectors. In the upconversion measurements, a photomultiplier (R7154P Hamamatsu, Hamamatsu City, Shizuoka Pref., Japan) adapted to the UV range, the so-called “solar blind”, was used so that the sample could be excited by a laser from the visible range without interfering with the measurement. In the range above 300 nm, the sensitivity of this photomultiplier drops rapidly, so the second band, located closer to the visible range, is “invisible” for this measurement. The peak of the more intense band is located at the wavenumber equal to 36,649 cm^−1^, while for the second band, it is at 32,465 cm^−1^ (with Stokes excitation, radiation of wavelength 160 nm). The difference between the peaks of these bands defined in this way is 4184 cm^−1^, which corresponds to the difference between the levels of the ^3^H_6_ and ^3^H_4_ multiplets, 5d→4f transitions for Pr^3+^ ions.

**Figure 11:**
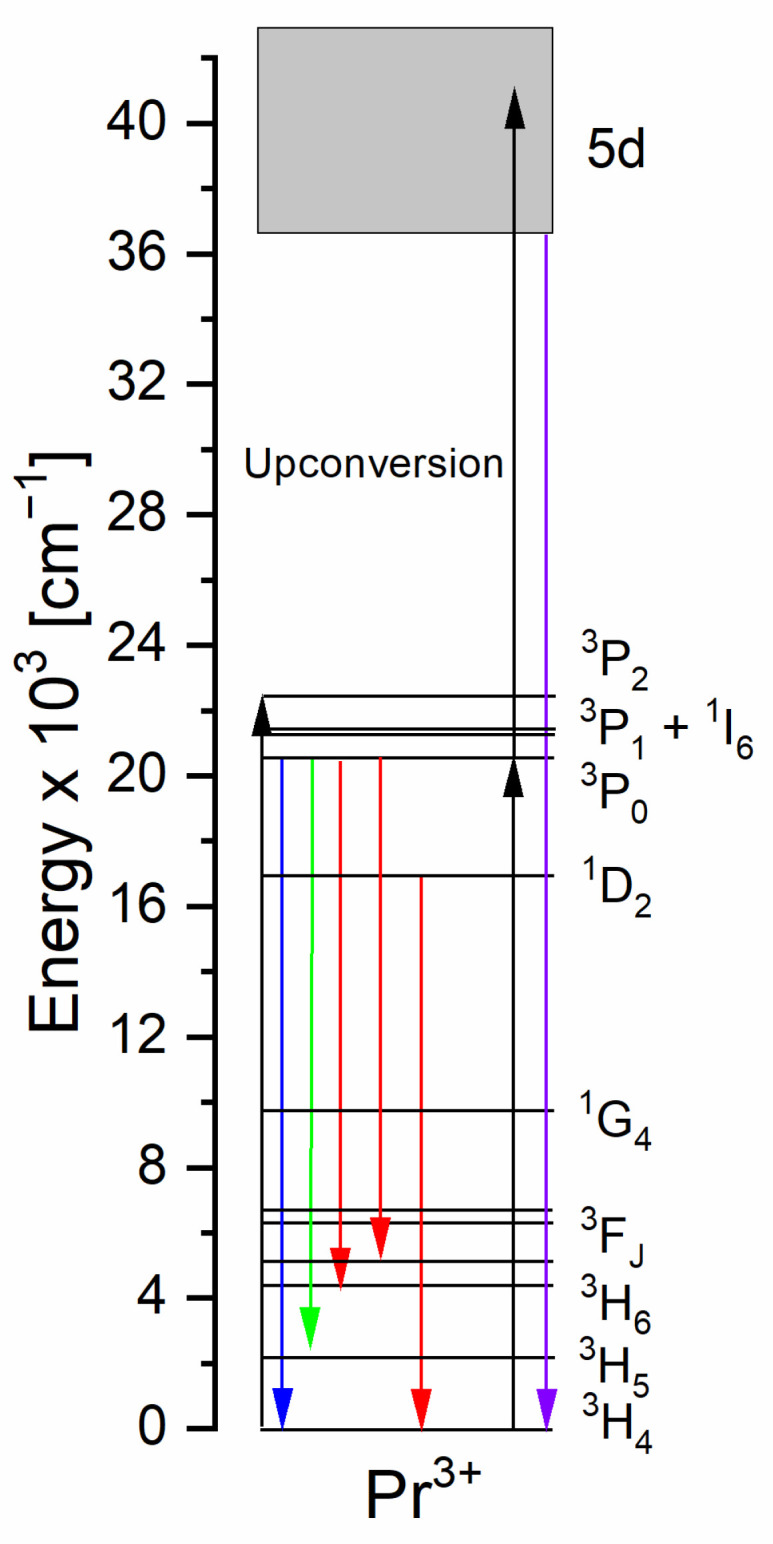
The excitation and emission mechanisms for the Pr^3+^ ions.

The mechanisms of the most important radiative processes are visualized on the energy level diagram for the praseodymium (III) ions (Figure 11). Blue radiation (e.g., at 444 nm, which is equal to 22,523 cm^−1^) pumps the ^3^P_J_ energy levels; then, mainly the ^3^P_0_ level emits blue, green, or red radiation. The blue radiation can also involve the upconversion process, where two lower-energy photons give one high-energy photon, from the 5d energy level, in the ultraviolet region.

**Figure 12:**
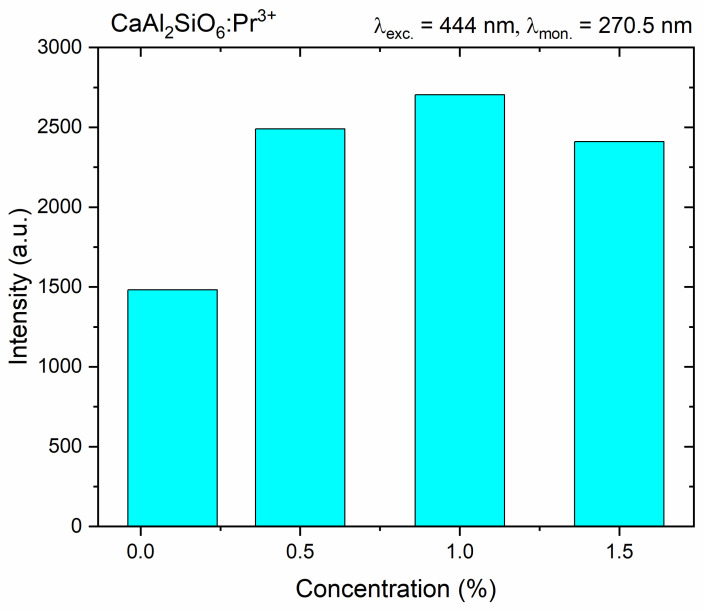
The visualization of the 5d→4f emission intensity obtained through the upconversion process for CaAl_2_SiO_6_:Pr^3+^ samples.

The results of the intensity of the anti-Stokes emission, excited by a blue laser at 444 nm and monitored at a wavelength of 270.5 nm, i.e., in the UVC region, are shown in Figure 12. With the increase in the Pr^3+^ ion content in the CASO matrix, the luminescence intensity increases due to the increase in the number of emission centers, with a maximum of 1% Pr^3+^ content. However, for a larger amount, 1.5% Pr^3+^, the intensity decreases due to non-radiative parasitic effects, which contribute to the luminescence quenching.

**Figure 13:**
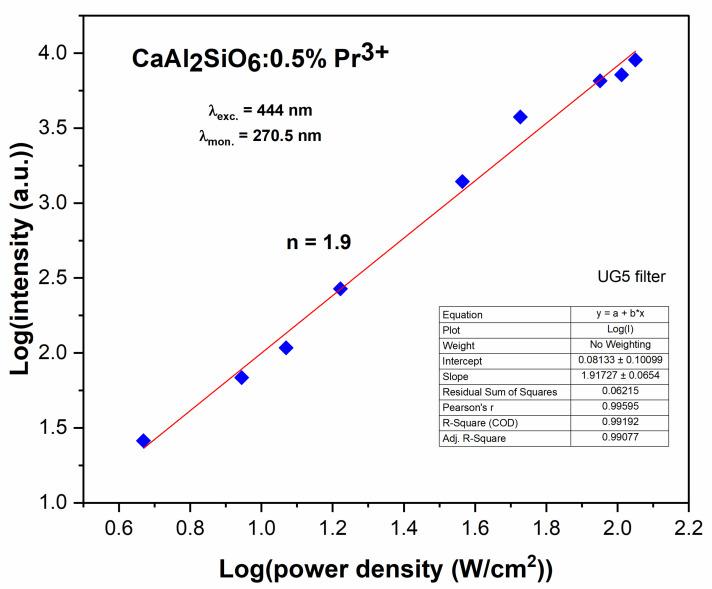
Power density dependence on the intensity of the 5d→4f upconversion emission for CaAl_2_SiO_6_:Pr^3+^ (excitation wavelength is 444 nm, and the monitored wavelength is 270.5 nm).

For the obtained upconversion in the UVC region, the influence of the laser radiation power density on the intensity of this emission was examined (Figure 13). This characteristic is in the log/log scale, because it is proportional to I ~ P^n^ [39], where I is intensity, P is laser power and n is a number of the pump photons required to excite the emitting level. The obtained characteristic can be fitted by the linear function, and *n* = 1.9, which means that two photons are involved in this process.

**Figure 14:**
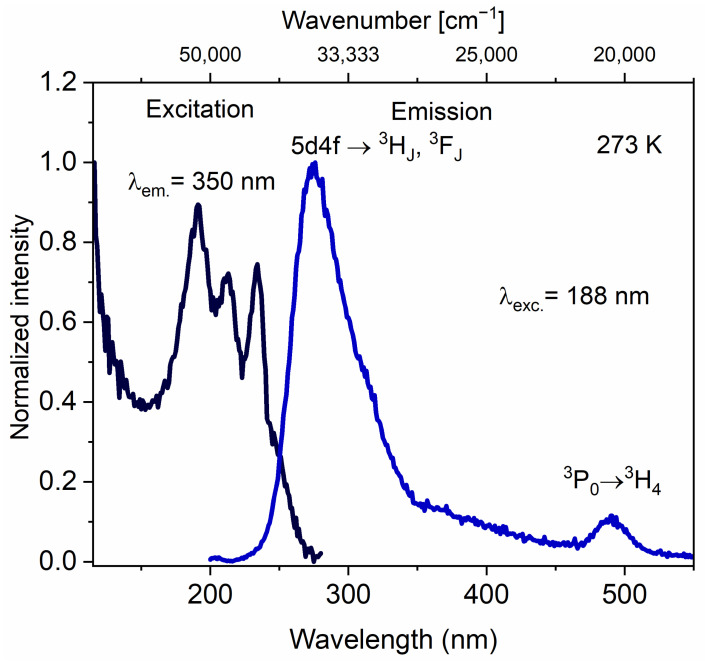
Excitation (λ_em._ = 350 nm) and emission (λ_exc._ = 188 nm) spectra of CaAl_2_SiO_6_:1.5% Pr^3+^ using synchrotron radiation.

The emission and excitation spectra of CaAl_2_SiO_6_:1.5% Pr^3+^ crystallites, obtained using synchrotron radiation, are characteristic of other Pr^3+^-activated silicate hosts [40] (Figure 14). The bands in the excitation spectrum from 150 to 270 nm correspond to the 4f^2^→5d^1^4f^1^ transition of the praseodymium ion [41]. Under 188 nm excitation, the emission spectrum is dominated in the 230–350 nm range, corresponding to the transition from 5d4f to ^3^H_J_ and ^3^F_J_ [42]. Additionally, the emission spectrum features a band with a maximum at 490 nm, attributed to the ^3^P_0_→^3^H_4_ transition of Pr^3+^. This occurs as a result of a non-radiative transition from the 5d electron configuration to the f configuration, often associated with significant Stokes shifts (ΔS) exceeding 3000 cm^−1^ [43]. However, according to theoretical predictions, the ideal hosts for blue-to-UVC upconversion should have a Stokes shift of less than 3000 cm^−1^ [44,45].

Based on the lowest-energy f–d excitation bands and the highest-energy d–f emission maxima (*E_em_*), the Stokes shift (∆S) for CASO was determined to be 3400 cm^−1^, explaining the observed emission in the visible region of the spectrum. Nevertheless, in CASO, the lowest energy level of 4f^1^5d^1^ for Pr^3+^ is quite low, namely 38,287 cm^−1^. This value was calculated using the following equation [20,46]:

(2) $$E_{fd}=E_{em}+1/2∆S,$$

where *E_fd_* is the lowest energy level. As a result, in the upconversion mechanism under 444 nm (22,522 cm^−1^) excitation, not only the ^3^P_0_ level but also the ^1^D_2_ level serve as intermediate states, making CaAl_2_SiO_6_ a prospective matrix for practical application.

**Figure 15:**
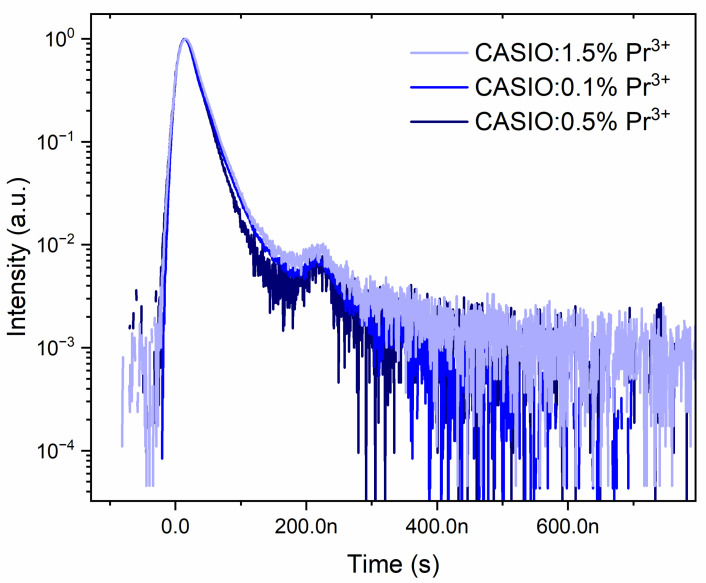
Decay profiles of upconversion emission under 443.4 nm pulsed excitation of CaAl_2_SiO_6_:*x*Pr^3+^.

The primary mechanism responsible for the luminescence of the praseodymium ion from the visible to the ultraviolet region is most often excited state absorption (ESA) [47,48]. All the decay profiles exhibit an additional peak of around 250 ns, attributed to a phenomenon known as ringing, which is common in this type of measurement [49,50]. The upconversion lifetime of the CASO phosphor was studied under excitation by a 443.4 nm pulsed laser (Figure 15). The upconversion decay time constants were determined based on the integral formula. The obtained decay times range from 24 to 21 ns, which is approximately equal to the 5d luminescence lifetime of the praseodymium ion [51]. The ESA mechanism is confirmed as the dominant process responsible for upconversion in this case.

## 3. Materials and Methods

### 3.1. Synthesis

CaAl_2_SiO_6_:Pr^3+^ nanoparticles were synthesized by the sol–gel method. Since the Pr^3+^ ion is close in size to the Ca^2+^ ion, it occupies the calcium position in the crystal lattice of CaAl_2_SiO_6_. To balance the charge, an appropriate amount of sodium ions was added to the aluminosilicate composition. The starting materials Ca(NO_3_)_2_*4H_2_O (99%, Alfa Aesar, Haverhill, MA, USA), Al(NO_3_)_3_*9H_2_O (99.999%, Sigma Aldrich, St. Louis, MO, USA), NaNO_3_ (99.8%, Sigma Aldrich, St. Louis, MO, USA), and Pr(NO_3_)_3_*6H_2_O (99.99%, Alfa Aesar, Haverhill, MA, USA) were dissolved in a mixture of 5 mL ethanol and 10 mL deionized water. The resulting clear solution was slowly poured into a stoichiometric amount of TEOS (Si(OC_2_H_5_)_4_ (99%, Sigma Aldrich, St. Louis, MO, USA) with vigorous stirring. The solution remained clear and gave no precipitate during the stirring process for 2 h at room temperature. To evaporate the solvent, the mixture was heated at 70 °C for 24 h. The resulting viscous gel was heated at a rate of 5 °C/min in an ambient atmosphere to 500 °C for 3 h then cooled to room temperature, ground in an agate mortar, and calcined at 1000 °C for 10 h. The following scheme shows the synthesis process:

2Al(NO_3_)_3_ + Ca(NO_3_)_2_ + xPr(NO_3_)_3_ + xNaNO_3_ + TEOS + C_2_H_5_OH →

Ca_1−2x_Al_2_Pr_x_Na_x_SiO_6_ + CO_2_↑ + H_2_O↑ + NO_2_↑

### 3.2. Measurements

X-ray powder diffraction (XRPD) measurements were collected using an X’Pert PRO X-ray diffractometer (Panalytical, Malvern, UK). The absorption spectra were recorded using a Cary 5000 UV-VIS-NIR spectrophotometer (Agilent Technologies, Santa Clara, CA, USA). Excitation, emission spectra, and decay time profiles were measured using an FLS1000 fluorescence spectrometer (Edinburgh Instruments, Livingston, UK), equipped with a 450 W xenon lamp. Emission characteristics were also determined using a femtosecond laser (Coherent Model “Libra”) that delivers a train of 100 fs pulses at a center wavelength of 800 nm and pulse energy up to 1 mJ with a repetition rate regulated up to 1 kHz. The lifetime of the short-living ^3^P_0_ level was obtained using the picosecond pulsed laser diode, operating at 450 nm. For the temperature-dependent emission spectra, the Hamamatsu (Hamamatsu City, Shizuoka Pref., Japan) PMA-12 detector with Linkam (Salfords, UK) THMS 600 Heating/Freezing Stage and a laser at 444 nm was used. Measurements in the UVC region were performed using a VUV McPherson spectrometer (McPherson, Chelmsford, MA, USA), equipped with a 150 W deuterium lamp. The upconversion emission was recorded on the McPherson spectrometer, using the excitation laser line at 444 nm. Excitation and emission spectra in ultraviolet range (in Figure 14) were recorded at SUPERLUMI (DESY, Germany) using a 2-meter McPherson monochromator and a Hamamatsu R6836 photomultiplier.

## 4. Conclusions

The new compounds of nanocrystalline CaAl_2_SiO_6_:Pr^3+^ aluminosilicates have been synthesized and measured for the first time. The investigation of the spectroscopic properties indicates that the blue, green, and red Stokes emissions of the Pr^3+^ occur in this crystal host. The Stokes emission in the visible range is observed mainly from the ^3^P_0_ or ^1^D_2_ energy levels to the ground state ^3^H_4_. The red emission of ^1^D_2_→^3^H_4_ is very stable even at very high temperatures.

The 5d→4f transition, mainly in the UVC but also in the UVB and UVA range, is observed by high-energy excitation in the UV range (Stokes emission) and by lower-energy excitation with visible light in the blue range (anti-Stokes emission). The UVC 5d→4f emission is much stronger than the 4f→4f luminescence. This is caused by the fact that the majority of the excitation energy is transferred directly to the 5d band, which does not lose much of this energy for transfer to the 4f praseodymium levels. Therefore, the UVC emission from the 5d level of Pr^3+^ ions in the CaAl_2_SiO_6_ matrix is much more efficient.

The investigated compounds are potentially useful for human health applications, because they possess characteristics that confirm their potential for upconversion emission in the UV range for disinfection purposes, to successfully destroy viruses and bacteria.

## Figures and Tables

**Table 1:** Lattice parameters, unit cell volume and calculated crystallite size of CaAl_2_SiO_6_:Pr^3+^ particles.

Sample	Lattice Parameters			Crystallite Size		Strain
	a (Å)	c (Å)	V (Å^3^)	SM (nm)	W-H (nm)	×10^−3^
CaAl_2_SiO_6_	9.9278	8.2172	701.39	68.8	82.0	1.33
CaAl_2_SiO_6_:0.1%Pr^3+^	9.9288	8.2397	703.46	71.3	119.5	0.81
CaAl_2_SiO_6_:0.5%Pr^3+^	9.9429	8.2325	704.84	70.3	86.6	1.04
CaAl_2_SiO_6_:1.0%Pr^3+^	9.9562	8.2394	707.32	65.6	70.9	1.71
CaAl_2_SiO_6_:1.5%Pr^3+^	9.962	8.2613	710.02	68.9	101.2	0.91

## Data Availability

The data presented in this study are available on request from the corresponding author.
